# Hybrid Organic Tandem Solar Cell Comprising Small-Molecule Bottom and Polymer:Fullerene Top Subcells Fabricated by Thin-Film Transfer

**DOI:** 10.1038/s41598-017-02181-6

**Published:** 2017-05-16

**Authors:** Yoonseok Ka, Hyejin Hwang, Changsoon Kim

**Affiliations:** 1 Program in Nano Science and Technology, Graduate School of Convergence Science and Technology, Seoul National University, Seoul, 08826 Republic of Korea; 2 Inter-University Semiconductor Research Center, Seoul National University, Seoul, 08826 Republic of Korea; 3 Advanced Institutes of Convergence Technology, Suwon, Gyeonggi, 16229 Republic of Korea

## Abstract

Multilayer structures involving solution-deposited polymer films are difficult to fabricate, not allowing for unrestricted designs of polymer-based optoelectronic devices required for maximizing their performance. Here, we fabricate a hybrid organic tandem solar cell whose top and bottom subcells have polymer:fullerene and small molecules active layers, respectively, by a solvent-free process based on transferring the polymer:fullerene layer from an elastomeric stamp onto a vacuum-deposited bottom subcell. The interface between small-molecule and transferred polymer:fullerene layers is void-free at the nanoscale, allowing for efficient charge transport across the interface. Consequently, the transfer-fabricated tandem cell has an open-circuit voltage (*V*_OC_) almost identical to the sum of *V*_OC_ values for the single-junction devices. The short-circuit current density (*J*_SC_) of the tandem cell is maximized by current matching achieved by varying the thickness of the small-molecule active layer in the bottom subcell, which is verified by numerical simulations. The optimized transfer-fabricated tandem cell, whose active layers are composed of poly[2,1,3-benzothiadiazole-4,7-diyl[4,4-bis(2-ethylhexyl)-4H-cyclopenta[2,1-b:3,4-b′]dithiophene-2,6-diyl]]:[6,6]-Phenyl-C_71_-butyric acid methyl ester and Di-[4-(N,N-di-p-tolyl-amino)-phenyl]cyclohexane:C_70_, has *V*_OC_ = 1.46 V, *J*_SC_ = 8.48 mA/cm^2^, a fill factor of 0.51, leading to the power-conversion efficiency of 6.26%, the highest among small molecule–polymer:fullerene hybrid tandem solar cells demonstrated so far.

## Introduction

Organic solar cells (OSCs) have potentials for lightweight, low-cost, and renewable energy sources^1–4^, but they have relatively low power-conversion efficiencies (PCEs) compared with those made of inorganic semiconductors such as silicon- or CIGS-based solar cells^5^. In order to increase the PCE of OSCs, many studies focusing on materials^6–8^, device structures^9, 10^, and fabrication techniques^11, 12^ have been reported. A particularly effective strategy is to use a tandem structure, where multiple single cells absorbing complementary spectral ranges are stacked^13–16^.

Depending on the materials used, organic tandem solar cells fall into three categories: polymer^13, 17–19^, small-molecule^20, 21^, and hybrid^22^ solar cells using both polymer and small-molecule materials. The attractive feature of hybrid organic tandem solar cells is that active materials can be chosen from a large number of candidates encompassing various polymers and small molecules. Therefore, using both polymer and small-molecule materials, it is easier to realize organic tandem cells with constituent subcells having complementary absorption spectra, which minimizes the thermalization loss if a device design is such that higher (and lower) energy photons are absorbed in the subcell with a higher (and lower) open-circuit voltage (*V*_OC_). Despite this advantage, the highest PCE of hybrid tandem solar cells reported so far is 4.8%^22^, while tandem devices based on polymers and small molecules, respectively, have achieved PCEs as high as 10.6%^15^ and 12%^23^. The relatively slow development in hybrid organic tandem solar cells is in part due to the following restriction on the device architecture. Since small-molecule materials can easily be degraded or dissolved by solvents used in spin-coating of polymers^24^, polymer layers must be deposited on substrates prior to the deposition of small molecules, which is typically achieved by thermal evaporation in vacuum. This leads to an optically undesirable configuration when the light is incident through the substrate side, if a low band gap polymer in a hybrid tandem solar cell is chosen to absorb longer wavelength photons^25^.

Here, we demonstrate a hybrid organic tandem solar cell comprising a polymer:fullerene-based top subcell and a small molecule-based bottom subcell using a thin-film transfer technique^12, 26–28^, where an active layer of the top subcell is transferred from an elastomeric stamp onto the bottom subcell. The bottom subcell with an active layer composed of (5,6)-Fullerene-C_70_ (C_70_) mixed with Di-[4-(N,N-di-p-tolyl-amino)-phenyl]cyclohexane (TAPC) mainly absorbs in the short wavelength region, while the top subcell, whose active layer is composed of poly[2,1,3-benzothiadiazole-4,7-diyl[4,4-bis(2-ethylhexyl)-4H-cyclopenta[2,1-b:3,4-b′]dithiophene-2,6-diyl]] (PCPDTBT) mixed with [6,6]-Phenyl-C_71_-butyric acid methyl ester (PC_70_BM), absorbs in the relatively longer wavelength region that cannot be absorbed in the bottom subcell (Supplementary Fig. S1). The value of *V*_OC_ for the tandem cell fabricated by the thin-film transfer technique is 1.46 V, which is almost identical to the sum of *V*_OC_’s of single-junction devices. The PCE of the tandem cell is 6.26%, which is, to the best of our knowledge, the highest among small molecule-polymer hybrid tandem solar cells demonstrated so far^22^. These results are attributed to the fact that the interface between the PCPDTBT:PC_70_BM and a small-molecule layer formed by thin-film transfer is defect-free at the nanoscale, as was confirmed by cross-sectional transmission electron microscopy (TEM). Our work shows that the thin-film transfer technique is capable of overcoming the restriction present in designing small molecule–polymer tandem solar cells, that is, the polymer subcell must be at the bottom, thereby allowing for the maximal utilization of materials space that small molecules and polymers offer in combination.

## Results and Discussion

**Figure 1:**
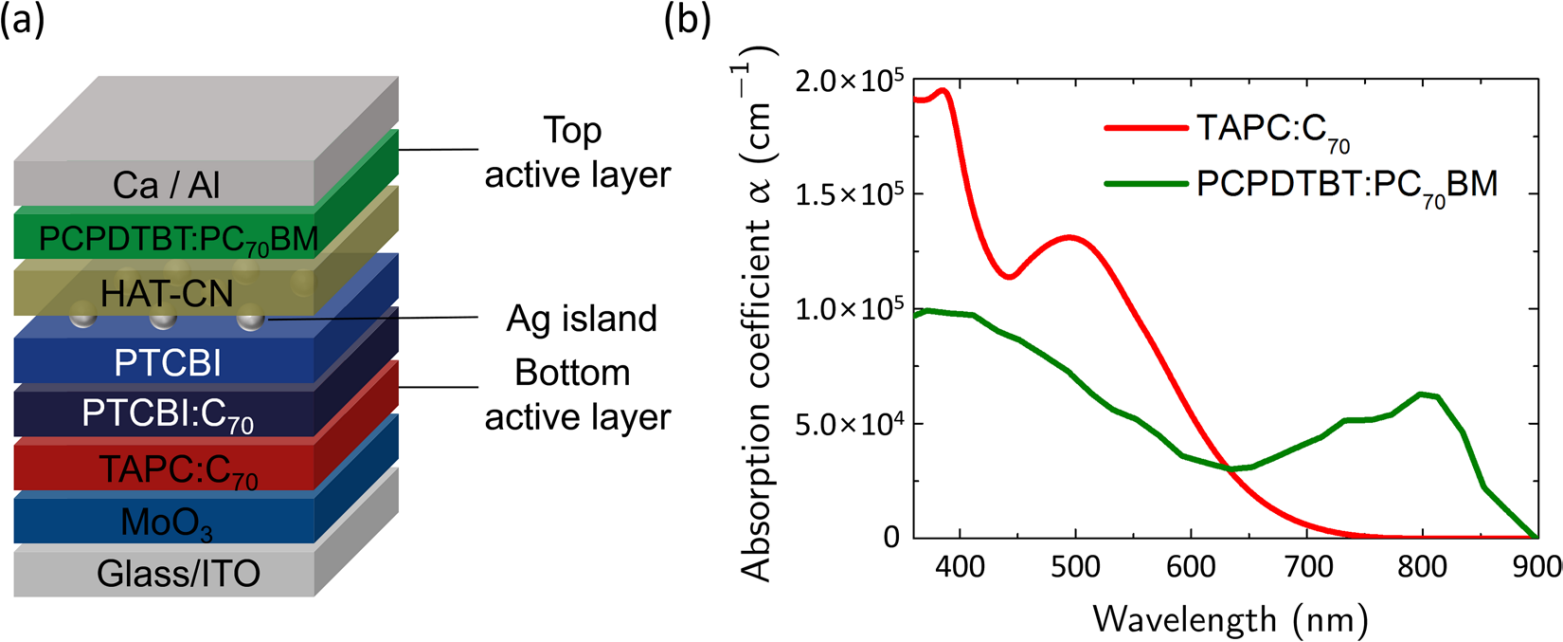
Device structure of transfer-fabricated tandem cells and absorption coefficients of active layers. (**a**) Device structure of a transfer-fabricated tandem cell whose top and bottom subcells have active layers composed of PCPDTBT:PC_70_BM and TAPC:C_70_ (**b**) Absorption coefficients of PCPDTBT:PC_70_BM (green) and TAPC:C_70_ (red) determined by spectroscopic ellipsometry.

Figure 1(a) shows a structure of hybrid organic tandem solar cells fabricated in this study comprising: glass/185 nm indium tin oxide (ITO)/2 nm MoO_3_/TAPC:C_70_/3 nm 3,4,9,10-perylenetetracarboxylic bisimidazole (PTCBI):C_70_/4 nm PTCBI/0.1 nm Ag/10 nm hexaazatriphenylene-hexacarbonitrile (HAT-CN)/90 nm PCPDTBT:PC_70_BM/20 nm Ca/100 nm Al. The absorption coefficients of TAPC:C_70_ and PCPDTBT: PC_70_BM films determined by spectroscopic ellipsometry [Fig. 1(b) and Supplementary Fig. S2] show that both layers are absorptive in the spectral region with wavelength λ less than 650 nm, while appreciable absorption of photons with *λ* > 700 nm can occur only in the PCPDTBT:PC_70_BM. Since the values for *V*_OC_ of typical TAPC:C_70_-based single-junction devices are ~ 0.85 V^14^, which is larger than those of PCPDTBT:PC_70_BM single-junction devices^29^ (*V*_OC_ ~ 0.60 V), in an optimized tandem cell where the thermalization loss is minimized, absorption of the shorter-wavelength photons (*λ* < 650 nm) must occur in the TAPC:C_70_. Therefore, the TAPC:C_70_ was chosen as the absorption layer of the bottom subcell, requiring that the PCPDTBT:PC_70_BM absorption layer of the top subcell must be deposited without degrading the underlying small-molecule layers. The PTCBI/Ag/HAT-CN trilayer is the interconnection layer (ICL) electrically connecting the two subcells: electrons photogenerated in the bottom subcell are transported through the PTCBI to recombine in the Ag islands with photogenerated holes transported from the top subcell through the HAT-CN layer. The PTCBI:C_70_ buffer layer was inserted between the TAPC:C_70_ and PTCBI layers to facilitate electron transport and thus increase the fill factor (FF)^30^.

**Figure 2:**
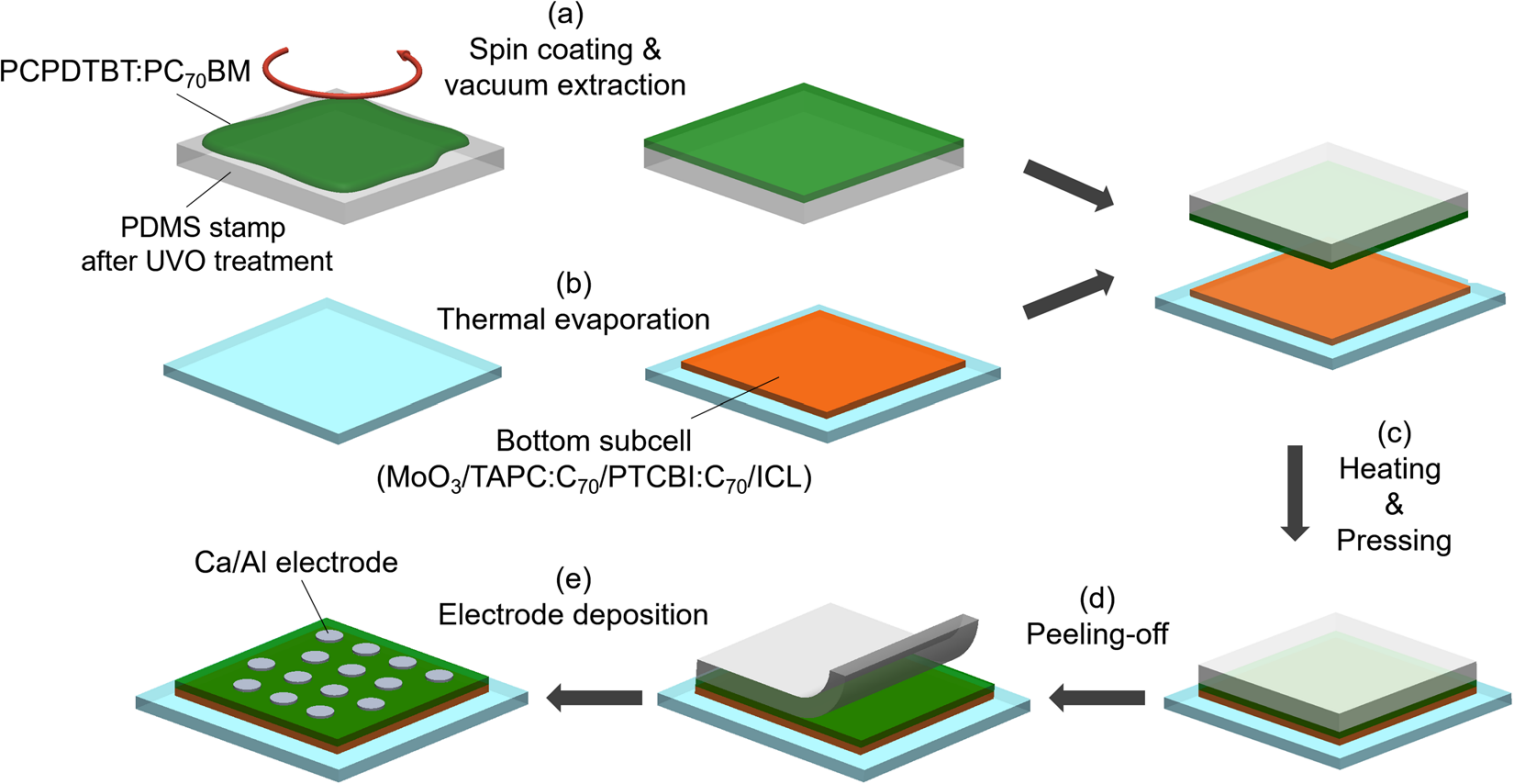
Schematic illustration of fabrication of hybrid organic tandem solar cells by the thin-film transfer technique. (**a**) Spin-coating of a PCPDTBT:PC_70_BM solution on a PDMS stamp and removal of a solvent and an additive. (**b**) Bottom subcell deposition on an ITO-coated glass substrate by thermal evaporation in vacuum. (**c**) Pressing of the stamp on the substrate with mild heating. (**d**) Peeling off the PDMS stamp from the substrate. (**e**) Deposition of the top electrodes.

The fabrication process of the hybrid organic tandem solar cell by thin-film transfer technique is schematically illustrated in Fig. 2. First, a PCPDTBT:PC_70_BM solution, prepared by mixing PCPDTBT and PC_70_BM in chlorobenzene (CB) with a 1,8-diiodooctane (DIO) additive, is spin-coated on a UV-ozone treated elasmoteric stamp made of poly(dimethylsiloxane) (PDMS) [Fig. 2(a)]. Immediately following the spin-coating, the PDMS stamp is stored in high vacuum (~10^−7^ Torr) for 1.5 h to remove the DIO additive, which is required to obtain a bulk heterojunction (BHJ) morphology with a high internal quantum efficiency (IQE)^31^. The immediate removal of the additive is also found to be crucial for successful transfer of the PCPDTBT:PC_70_BM BHJ layer onto the bottom subcell, since a slower drying process, such as commonly used solvent annealing^32^, causes the PDMS stamp to swell, which leads to diffusion of PCPDTBT:PC_70_BM into the stamp. In parallel, a small molecule-based bottom subcell is prepared by sequentially depositing MoO_3_, TAPC:C_70_, and PTCBI:C_70_ layers on a UV-ozone treated ITO-coated glass substrate by thermal evaporation in vacuum, followed by deposition of the ICL consisting of PTCBI/Ag/HAT-CN, also by thermal evaporation in vacuum [Fig. 2(b)]. Next, the stamp is pressed onto the substrate with mild heating [Fig. 2(c)], and the PCPDTBT:PC_70_BM film is transferred onto the ICL as the stamp is peeled off from the substrate [Fig. 2(d)]. Deposition of the top electrode composed of Ca/Al through a shadow mask by thermal evaporation in vacuum completes the fabrication of the hybrid organic tandem solar cell [Fig. 2(e)]. In general, an optimal BHJ morphology of a polymer:fullerene derivative layer is formed by inducing an appropriate degree of phase separation between the constituent materials^33^. Since the phase separation process depends critically on experimental variables such as spin-coating speed, fullerene derivative concentration, and solvent evaporation rate^33^, a BHJ morphology with a high IQE is difficult to obtain over a broad range of thicknesses^29^. Therefore, the thickness of the PCPDTBT:PC_70_BM layer was fixed at 90 nm, where a processing condition described in the Method Section yields a BHJ layer with a high IQE. In contrast, since varying the layer thickness without significantly affecting the BHJ morphology is relatively easier for a vacuum-deposited TAPC:C_70_ film, the thickness of that layer *t* was varied so that four sets of tandem devices with *t* = 90, 120, 160, and 200 nm, respectively, were fabricated. In addition, a hybrid organic tandem solar cell where its PCPDTBT:PC_70_BM layer was deposited by spin-coating, instead of thin-film transfer, and where all other layers were identical to the corresponding layers in the transfer-fabricated devices was prepared for comparison.

**Figure 3:**
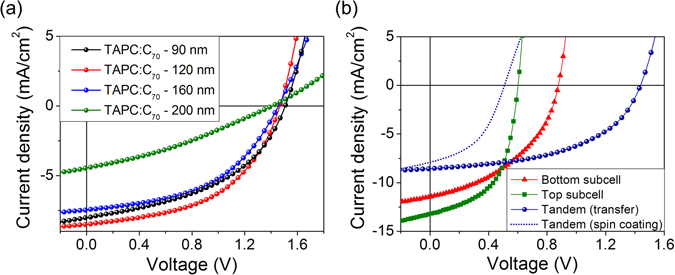
Current density vs voltage (*J*–*V*) characteristics of single-junction and tandem solar cells. (**a**) *J*–*V* characteristics of transfer-fabricated organic tandem solar cells with varying TAPC:C_70_ thickness. (**b**) *J*–*V* characteristics of transfer-fabricated and spin-coated tandem cells with 120-nm-thick TAPC:C_70_ layers, and two single-junction cells whose active layers are TAPC:C_70_ and PCPDTBT:PC_70_BM, respectively.

Figure 3(a) shows the current density versus voltage (*J*–*V*) characteristics of the hybrid organic tandem solar cells with different *t*, whose PCPDTBT:PC_70_BM layer was deposited by thin-film transfer, measured under simulated 1 sun AM 1.5 G illumination. The device parameters, such as *V*_OC_, short-circuit current density *J*_SC_, FF, and PCE, are listed in Table 1. As *t* increases from 90 to 120 nm, *J*_SC_ increases from 8.04 to 8.48 mA/cm^2^, and a further increase in *t* decreases *J*_SC_. This result indicates, as discussed later more quantitatively, that (i) at *t* = 90 nm, *J*_SC_ of the tandem cell is limited by insufficient optical absorption in the bottom subcell, (ii) near *t* = 120 nm, the currents generated in both subcells are matched to each other, thereby maximizing *J*_SC_ of the tandem device, and (iii) *t* > ~120 nm is optically too thick, meaning that the bottom subcell absorbs too many photons so that *J*_SC_ of the tandem cell is limited by the top subcell. As a result of the short-circuit current matching, the device with *t* = 120 nm (red circles) has the highest *J*_SC_ of 8.48 mA/cm^2^, with *V*_OC_ = 1.46 V and FF = 0.51, leading to the highest PCE of 6.26%. To the best of our knowledge, this is the highest PCE among small molecule–polymer hybrid organic tandem solar cells reported so far^22, 33, 34^. The device with *t* = 200 nm (green circles) suffers not only from a large imbalance between the subcell currents but also from a substantially decreased FF arising from increased series resistance. Consequently, it has the lowest PCE of 1.99%. Figure 3(b) compares the *J*–*V* curve of the tandem device with the highest PCE (the device with *t* = 120 nm, blue circles) with that of a single-junction device whose active layer is TAPC:C_70_ (red triangles) or PCPDTBT:PC_70_BM (green squares). The structures of the single-junction devices are: glass/185 nm ITO/2 nm MoO_3_/120 nm TAPC:C_70_/3 nm PTCBI:C_70_/4 nm PTCBI/100 nm Ag, and glass/185 nm ITO/10 nm HAT-CN/90 nm PCPDTBT:PC_70_BM/20 nm Ca/100 nm Al. The value of *V*_OC_ for the tandem cell (1.46 V) is almost identical to the sum of *V*_OC_ values for single-junction devices (1.48 V), indicating that holes photogenerated in the top subcell are readily transported across the PCPDTBT:PC_70_BM–HAT-CN interface formed by thin-film transfer and recombine with electrons transported from the bottom subcell. Also shown in Fig. 3(b) is the *J*–*V* curve of a tandem device whose device structure was identical to that of the transfer-fabricated tandem device, except for its PCPDTBT:PC_70_BM layer deposited by spin-coating, instead of thin-film transfer (dotted line). For the spin-coated device, *V*_OC_ is significantly decreased to 0.51 V, indicating that the series connection between the top and bottom subcells was not properly made in this case.

**Table 1:** Photovoltaic parameters of hybrid organic tandem solar cells with different thicknesses of the bottom active layer (TAPC:C_70_) and single-junction cells whose active layers are TAPC:C_70_ or PCPDTBT:PC_70_BM.

	TAPC:C_70_ Thickness (nm)	*J*_SC_ (mA/cm^2^)	*V*_OC_ (V)	FF	PCE (%)
Single-junction cell (TAPC:C_70_)	120	11.6	0.89	0.42	4.39
Single-junction cell (PCPDTBT:PC_70_BM)	·	13.2	0.59	0.55	4.26
Tandem cell (transfer-fabricated)	90	8.04	1.5	0.46	5.49
	120	8.48	1.46	0.51	6.26
	160	7.44	1.45	0.48	5.22
	200	4.45	1.4	0.32	1.99
Tandem cell (spin-coated)	120	7.9	0.51	0.45	1.82

**Figure 4:**
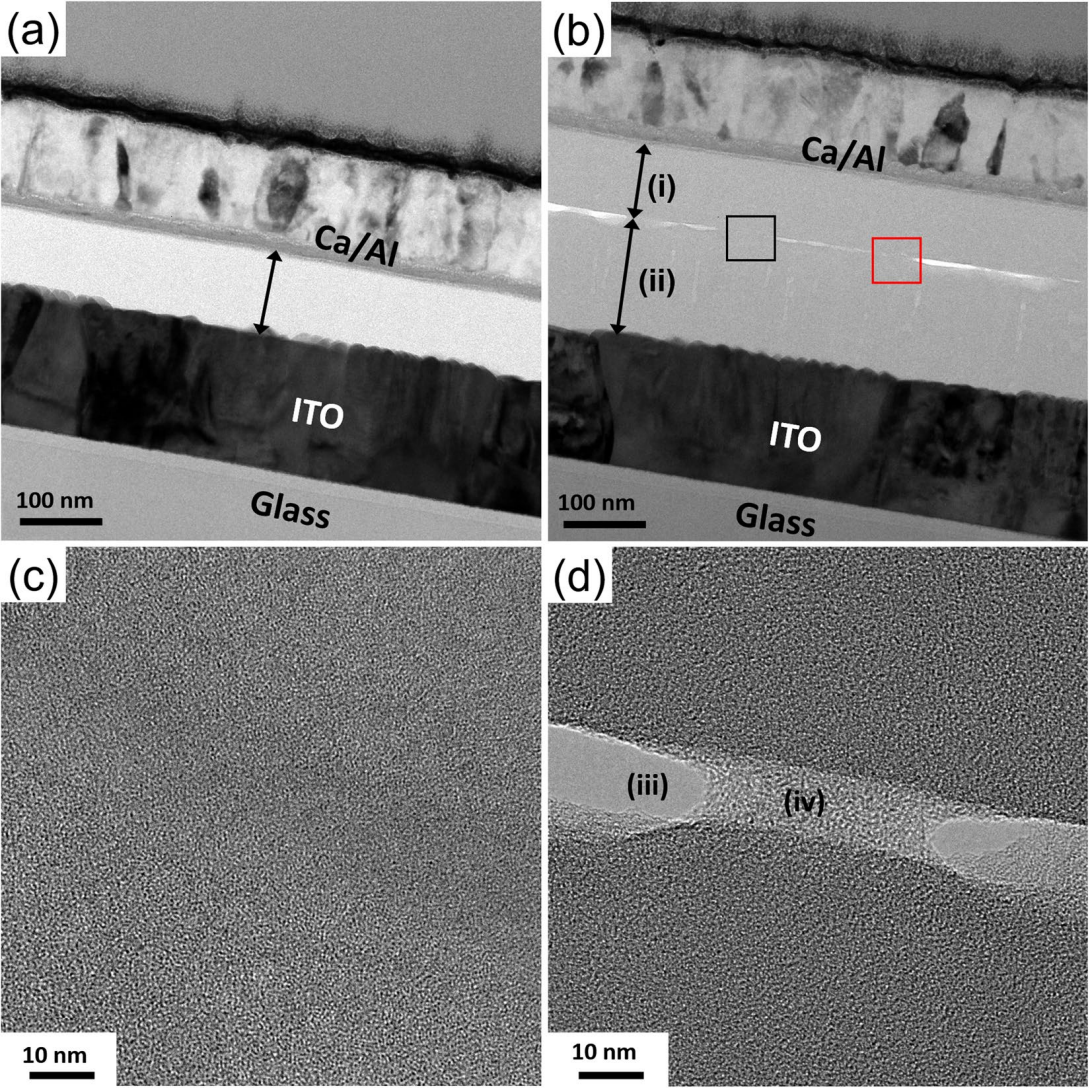
Cross-sectional transmission electron microscope (TEM) images of hybrid organic tandem solar cells. Cross-sectional TEM image of (**a**) spin-coated and (**b–d**) transfer-fabricated tandem solar cells. (**c**) and (**d**) are the images of the regions enclosed by black and red squares in (**b**), respectively. For the transfer-fabricated device, thicknesses of the transferred PCPDTBT:PC_70_BM layer (i) and the thermally evaporated small-molecule layers (ii) were unchanged from the designed values. The features (iii) and (iv) in (**d**) were generated during focused-ion beam milling used in sample preparation.

In Fig. 4, the cross-sectional TEM images of the transfer-fabricated hybrid organic tandem solar cell are compared with those of the spin-coated device. In the case of the spin-coated device, the total thickness of the layers between the two electrodes, which are supposed to be the small-molecule layers for the bottom subcell, the ICL layer, and the PCPDTBT:PC_70_BM layer, is found to be ~90 nm, as indicated by an arrow in Fig. 4(a). This is much smaller than the designed value of 230 nm: the spin-coating condition was the same as that used to obtain 90-nm-thick PCPDTBT:PC_70_BM films on PDMS stamps in Fig. 2(a). As expected, this result is due to the penetration of the solvent through the ICL and the bottom subcell, and the resulting intermixing of the constituent layers, leading to a very poor device performance as shown in Fig. 4(a) and Table 1. In contrast, in the transfer-fabricated device, the total thickness of the tandem device was unchanged from the designed value, with the transfer-fabricated PCPDTBT:PC_70_BM (i) and thermally evaporated small-molecule (ii) layers marked by arrows in Fig. 4(b), which implies that the small-molecule layers were not damaged by the thin-film transfer process. Furthermore, the morphological quality of the HAT-CN–PCPDTBT:PC_70_BM interface formed by thin-film transfer is remarkably high so that it cannot be identified even in a high-resolution image [Fig. 4(c)] of the area enclosed by a black square in Fig. 4(b). This is in line with our previous report where a MoO_3_–polymer:fullerene interface that is void-free at the nanoscale has been formed by transferring the polymer:fullerene layer onto the MoO_3_ layer^27^. Figure 4(d) is a high-resolution image of the area in Fig. 4(b) enclosed by a red square, showing voids (iii) and a region (iv) that is brighter than the surrounding. These features were determined to have been generated by damage incurred during focused-ion beam milling used in sample preparation^35^, since the *J*–*V* curve with characteristics typical of a tandem device with good electrical connection between the subcells, as shown in Fig. 3, cannot be expected, if the features, which are observed in large areas at the interface as shown in Fig. 4(b), had been caused by the thin-film transfer process and therefore had been present in the device before the *J*–*V* measurement. It may be argued that the good *J*–*V* characteristic shown in Fig. 3 can still be obtained in the presence of the features if photogenerated electrons and holes migrate to void-free regions to recombine with each other. This possibility, however, is ruled out since the lateral dimensions of the regions with the features are comparable to the thickness of the device [Fig. 4(b)], and therefore a non-negligible loss in voltage or current must have been observed if they had not been caused by damage from the focused-ion beam process.

**Figure 5:**
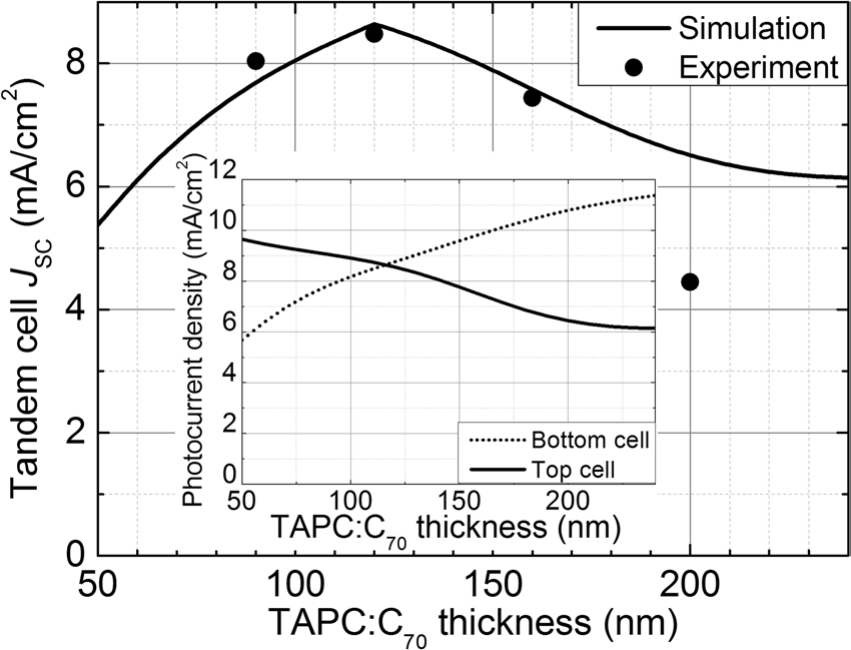
Calculated and measured short-circuit current densities (*J*_SC_) of the transfer-fabricated tandem devices with different TAPC:C_70_ thicknesses (*t*). The calculated (line) and measured (circle) values of *J*_SC_ for the transfer-fabricated tandem device shown in Fig. 1 as functions of *t*. The inset shows the photocurrent densities of the top (solid line) and bottom (dotted line) subcells as functions of *t*.

The dependency of *J*_SC_ of the transfer-fabricated device on *t*, summarized in Table 1, can be quantitatively explained by numerical simulations. To obtain simulated *J*_SC_, we first calculated the optical absorption efficiency of each active layer – the probability that a photon incident on the tandem cell is absorbed in that active layer – using the transfer-matrix method^36^. Next, for simplicity both subcells were assumed to have a common, wavelength-independent IQE that does not vary with the active layer thicknesses. The EQE of each active layer – the probability that a photon incident on the tandem cell leads to separated charge carriers resulting from absorption in that active layer – was then given by the product of its absorption efficiency and IQE. The solar-spectrum-weighted integral of EQE of each active layer yielded the photocurrent density of each subcell, the smaller of which was taken as an estimate of *J*_SC_ of the tandem cell. Figure 5 shows the experimental (circle) and simulated (line) values of *J*_SC_ as a function of *t*, where the IQE, regarded as a fitting parameter, was determined to be 0.76 to minimize the total error between the experiment and simulation for *t* = 90, 120, and 160 nm. The data point at *t* = 200 nm was excluded in the fitting, since a decrease in IQE due to poor charge collection is often observed for a thick BHJ layer^37–39^. Except for *t* = 200 nm, at which the IQE is expected to be smaller than 0.76, the three data points are well fit by the simulation. In particular, the location of the maximum *J*_SC_ for the simulated case agrees well with that for the experiment, meaning that our simulation – although not useful in accurately predicting the actual values of *J*_SC_ due to assumptions made for simplicity – can be used as a simple yet effective guideline for maximizing *J*_SC_ of transfer-fabricated tandem solar cells. The inset of Fig. 5 shows that at *t* = 120 nm the photocurrents of the top (solid line) and bottom (dotted line) subcells are matched, maximizing *J*_SC_, and that as *t* increases (or decreases) from 120 nm, *J*_SC_ is limited by insufficient absorption in the top (or bottom) subcell.

## Conclusion

We have fabricated small molecule–polymer hybrid organic tandem solar cells whose active layers are based on PCPDTBT:PC_70_BM and TAPC:C_70_. By examining optical absorption spectra of both layers and values of *V*_OC_ expected from these donor–acceptor pairs, the PCPDTBT:PC_70_BM- and TAPC:C_70_-based subcells were determined to be located at the top and bottom, respectively, to maximize the PCE. This device configuration makes inapplicable the conventional spin-coating method for polymer deposition, due to damage to the underlying small-molecule layers by a liquid solvent used in spin-coating. We, therefore, have employed a solvent-free method where a PCPDTBT:PC_70_BM layer deposited on an elastomeric stamp is transferred onto a small-molecule bottom subcell. Cross-sectional TEM images show that the interface between PCPDTBT:PC_70_BM and small-molecule layers formed by thin-film transfer is remarkably intimate, leading to *V*_OC_ of the tandem device almost equaling the sum of *V*_OC_ of the two subcells. A tandem device optimized by varying the thickness of the TAPC:C_70_ layer has *J*_SC_ = 8.48 mA/cm^2^ and PCE = 6.26%, the latter being the highest among small molecule–polymer hybrid tandem solar cells reported so far. Since the thin-film transfer technique allows for the maximal utilization of materials choice that small molecules and polymers offer in combination, with further efforts, it may potentially realize commercially viable, highly efficient small molecule–polymer tandem solar cells.

## Methods

All organic materials were purchased and used without further purification. Poly[2,1,3-benzothiadiazole-4,7-diyl[4,4-bis(2-ethylhexyl)-4H-cyclopenta[2,1-b:3,4-b′]dithiophene-2,6-diyl]] (PCPDTBT) and fullerene derivative [6,6]-phenyl C_71_-butyric acid methyl ester (PC_70_BM) were purchased from 1-material, Inc. and Nano-C, Inc., respectively. (5,6)-Fullerene-C_70_ (C_70_) and Di-[4-(N,N-di-p-tolyl-amino)-phenyl]cyclohexane (TAPC) were purchased from Lumtec, while hexaazatriphenylene-hexacarbonitrile (HAT-CN) and 3,4,9,10-perylenetetracarboxylic bisimidazole (PTCBI) were purchased from Jilin OLED Material Technology.

All devices were fabricated on ITO-coated glass substrates (15 Ω/sq., 25 mm by 25 mm), which were sequentially cleaned with detergent, de-ionized water, acetone, and isopropyl alcohol, followed by baking at 200 °C for 10 min in a vacuum oven prior to film depositions. All layers except the PCPDTBT:PC_70_BM were deposited by thermal evaporation in vacuum (~10^−7^ Torr). Deposition rates were ~1 Å/s except for the 0.1-nm-thick Ag “layer” in the ICL and the Ca layer in the top electrodes, whose deposition rates were 0.1 Å/s and 0.2 Å/s, respectively. The TAPC:C_70_ layers were formed by doping C_70_ layers with TAPC at 5 wt.% by co-evaporation. Also, the PTCBI:C_70_ layers were deposited by co-evaporating PTCBI and C_70_ in a volume ratio of 1:1. Device areas were defined by patterning of top metallic electrodes achieved by metal evaporation through shadow masks with 2-mm-diameter circular openings. The PCPDTBT:PC_70_BM solution was prepared by dissolving 7.5 mg of PCPDTBT and 26.9 mg of PC_70_BM into a mixture of 0.03 ml DIO and 0.97 ml CB, with a magnetic stirring bar at 70 °C for 6 h^17^, and was spin coated at 2000 rpm for 20 s to obtain 90-nm-thick PCPDTBT:PC_70_BM layers on PDMS stamps. PDMS stamps were prepared by following a process described elsewhere^12^. Pressing of PCPDTBT:PC_70_BM-coated PDMS stamps onto substrates to transfer PCPDTBT:PC_70_BM films onto HAT-CN layers for fabrication of the tandem and single-junction devices was performed using a custom-built apparatus in a N_2_ glove box, as described elsewhere^27^. To ensure that a conformal, intimate contact at the PCPDTBT:PC_70_BM–HAT-CN interface was obtained throughout the substrate area, a pressure of 21.7 kgf/cm^2^ was applied for 3 min while heating the samples at 100 °C.

The *J*–*V* characteristics were measured using a source meter (2400, Keithley) and a solar simulator (PEC-L01, Peccell Technologies) calibrated with a standard silicon solar cell (BS-520BK, Bunkoukeiki). The cross-sectional images of the spin-coated and transfer-fabricated tandem solar cells were obtained using a TEM (JEM-2100F, JEOL) and the samples for the TEM measurements were prepared using a focused ion beam instrument (Quanta 3D FEG, FEI).

## Electronic supplementary material

Supplementary Information
