# Supplementary material for: Hybrid Organic Tandem Solar Cell Comprising Small-Molecule Bottom and Polymer:Fullerene Top Subcells Fabricated by Thin-Film Transfer
Source: Sci Rep. 2017 May 16;7:1942. doi: 10.1038/s41598-017-02181-6 (PMC5434005; doi:10.1038/s41598-017-02181-6)
Supplement: Supplementary file 1 — Supplementary Information [file 41598_2017_2181_MOESM1_ESM.pdf]

Hybrid Organic Tandem Solar Cell Comprising Small-Molecule Bottom and Polymer:Fullerene Top Subcells Fabricated by Thin-Film Transfer

Supplementary Information

Yoonseok Ka[1], Hyejin Hwang[1] & Changsoon Kim[1, 2, 3,*]

[1]Program in Nano Science and Technology, Graduate School of Convergence Science and Technology, Seoul National University, Seoul 08826, Republic of Korea.

[2]Inter-University Semiconductor Research Center, Seoul National University, Seoul 08826, Republic of Korea.

[3]Advanced Institutes of Convergence Technology, Suwon, Gyeonggi 16229, Republic of Korea.

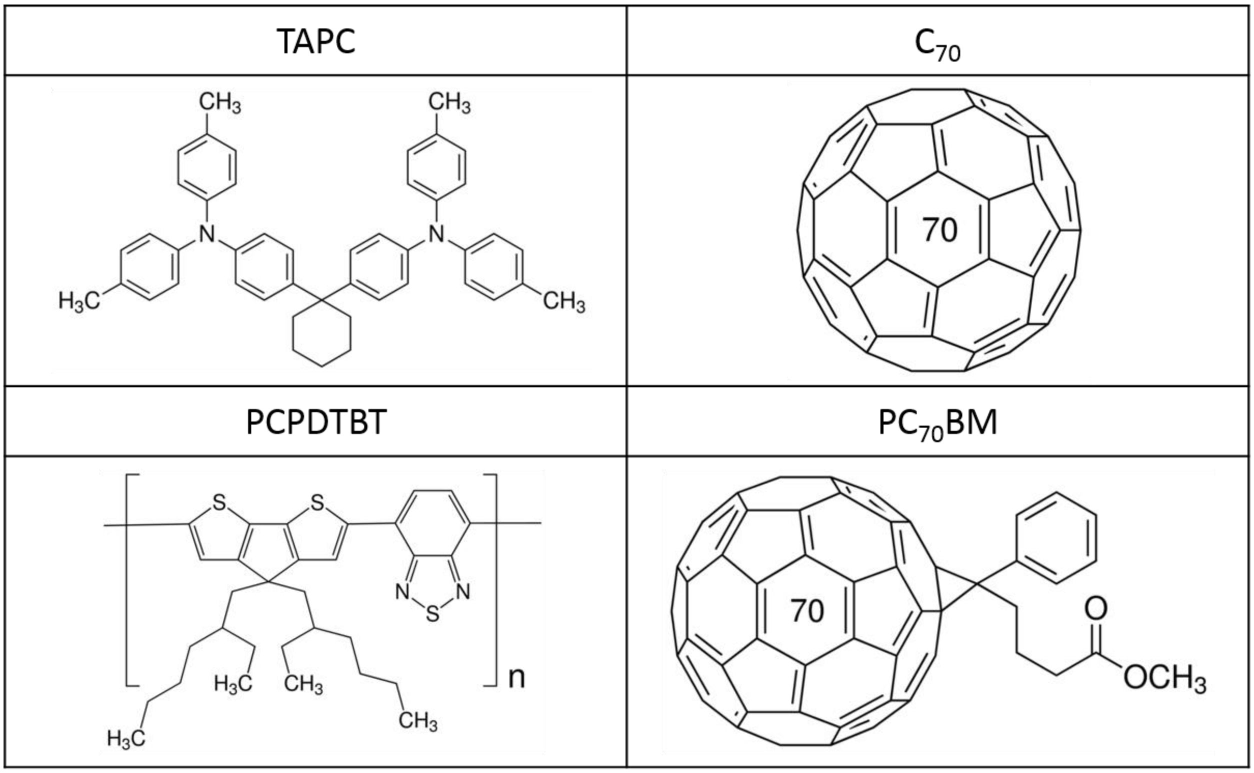

Figure S1. Chemical structures of materials used in the active layers.

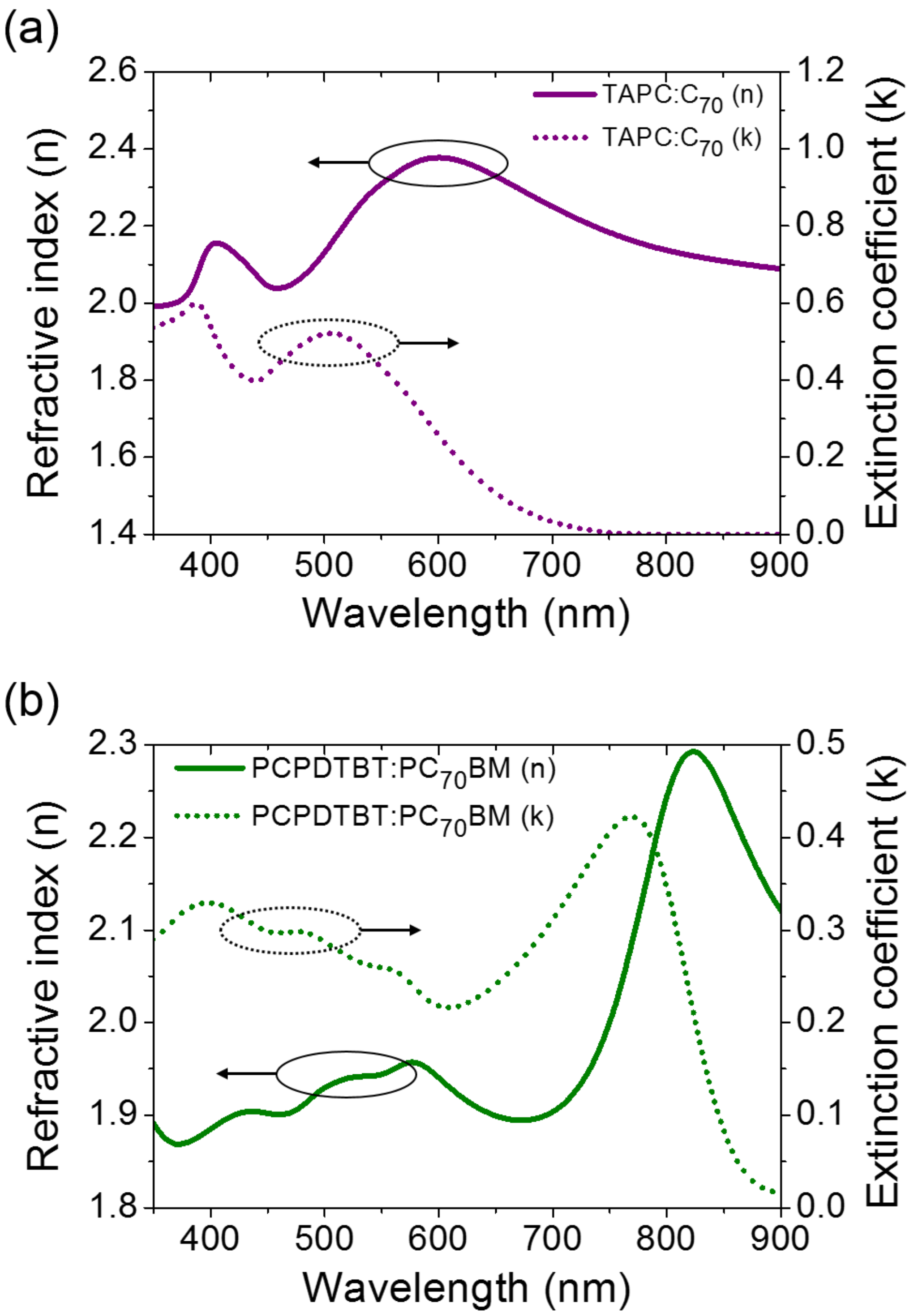

Figure S2. Refractive index (solid lines) and extinction coefficient (dotted lines) of (a) TAPC:C_{70} and (b) PCPDTBT:PC_{70}BM layer.
